# Examining Subjective Aging through the Lens of the Caregiver Experience

**DOI:** 10.1093/geroni/igab046.2922

**Published:** 2021-12-17

**Authors:** Kristen Hardin-Sigler, Kaitlin Grelle, Rebecca Deason

**Affiliations:** Texas State University, San Marcos, Texas, United States

## Abstract

Caregivers of individuals with Alzheimer’s disease and related dementia (ADRD) often experience burden that has been associated with poor physical and psychological health outcomes (Andren & Elmstahl, 2007; Zimmerman et al., 2018). However, very little research investigates how the caregiving experience may impact an individual’s subjective aging experience. Various aspects of subjective aging have been implicated in health outcomes and memory function (Brothers et al., 2017; Stephan, Sutin, Caudroit, & Terracciano, 2016). The purpose of this study was to investigate the differences in perceptions of subjective aging between caregivers and non-caregivers. Participants (N = 185) completed a survey assessing several aspects of subjective aging, including subjective age, or how old an individual feels, memory function, well-being, attitudes towards aging, and aging stereotypes. A series of independent t-tests indicated that there were significant differences between groups on subjective age (p = .013), and subjective memory function (p = .013). Caregivers (n = 93) reported feeling significantly older than their chronological age, reported significantly more subjective memory complaints, and also reported poorer subjective memory function when compared to the non-caregiver (n = 92) control group. Previous literature does suggest that older subjective age ratings are associated with poor subjective memory function, so these results are not necessarily surprising. However, these results suggest that caregiving for individuals with ADRD may negatively impact caregivers’ perceptions of their own aging experience, but not necessarily their perceptions about aging in general.

